# Risk factors of in-stent restenosis after carotid angioplasty and stenting: long-term follow-up study

**DOI:** 10.3389/fneur.2024.1411045

**Published:** 2024-08-08

**Authors:** Sangil Park, Bum Joon Kim, Hye-Yeon Choi, Dae-Il Chang, Ho Geol Woo, Sung Hyuk Heo

**Affiliations:** ^1^Department of Neurology, Uijeongbu Eulji Medical Center, Eulji University School of Medicine, Uijeongbu, Republic of Korea; ^2^Department of Neurology, Kyung Hee University Hospital, Kyung Hee University College of Medicine, Seoul, Republic of Korea; ^3^Department of Neurology, Asan Medical Center, College of Medicine, University of Ulsan, Seoul, Republic of Korea; ^4^Department of Neurology, Kyung Hee University Hospital at Gangdong, Kyung Hee University College of Medicine, Seoul, Republic of Korea; ^5^Department of Neurology, Gangnam Severance Hospital, Yonsei University College of Medicine, Seoul, Republic of Korea

**Keywords:** angioplasty, carotid stenosis, restenosis, stents, ultrasonography

## Abstract

**Background:**

After carotid artery angioplasty with stenting (CAS), it is unclear which risk factors are related to long-term outcomes, including in-stent restenosis (ISR). This study aimed to assess the factors associated with restenosis after CAS with a median follow-up of 35.7 months.

**Materials and methods:**

Patients who underwent CAS from January 2013 to December 2018 were included if they had symptomatic or asymptomatic carotid artery stenosis. The carotid Doppler ultrasonography (CDU) was followed up after the procedure. We defined at least 50% restenosis using the criteria that the internal carotid artery (ICA) peak systolic velocity (PSV) was greater than 224 cm/s or the ICA to common carotid artery PSV ratio was higher than 3.4. The risk factors for ISR were also assessed.

**Results:**

Of the 189 patients, 122 had symptomatic carotid artery stenosis, and 67 had asymptomatic carotid artery stenosis. Patients were evaluated by CDU for a median of 35.7 months (interquartile range 19.5 to 70.0). Kaplan–Meier analysis showed that the longest time to ISR was 39 months, and ISR-free was better in the asymptomatic CAS group. In all groups, ISR was independently associated with current smoker [adjusted odds ratio (aOR), 3.425; 95% confidence interval (CI), 1.086 to 10.801] and elevated ICA PSV at baseline (aOR, 1.004; 95% CI, 1.001 to 1.007).

**Conclusion:**

Independent risk factors for ISR in the CAS group included current smoking and elevated ICA PSV at baseline. In the symptomatic CAS group, alcohol was independently associated with the ISR. ISR did not occur after 39 months from the CAS procedure in our study patients. Future studies with extended follow-up are necessary to fully understand the long-term outcomes of CAS.

## Introduction

Cerebrovascular disease was a major cause of death and disability (1). Approximately 8% to 25% of ischemic strokes are related to carotid artery stenosis (2). To prevent ischemic stroke, treatments such as carotid angioplasty and stenting (CAS) insertion are recommended for significant carotid stenosis (3). In addition to preventing stroke, reopening carotid stenosis enhances the quality of life by reducing vertigo and improving cognition (4). Therefore, stent insertion has effects beyond stroke prevention over time.

According to previous reports, ischemic stroke in asymptomatic patients with carotid stenosis of more than 50% is reported in 10%–15% of the cases (5). According to guidelines published by the American College of Cardiology (ACC) and American Heart Association (AHA), CAS is recommended for symptomatic patients with stenosis greater than 50% identified by catheter angiography (6). The AHA and American Stroke Association (ASA) advise preventive CAS as the primary stroke prevention method if there is 70% asymptomatic carotid stenosis on carotid Doppler ultrasonography (CDU) (7). In a recent systematic study, CAS did not significantly enhance the risk of perioperative death or stroke in patients with asymptomatic carotid artery stenosis compared to those with carotid endarterectomy (CEA) (8). However, owing to a lack of available data at the time, an investigation of long-term efficacy was not possible (9). Unfortunately, there is always controversy regarding the optimal treatment strategy for patients with carotid disease (10).

Recent advancements in engineering tools have significantly impacted the field of medical image processing and simulations, particularly in predicting blood hemodynamics (11). These tools enable precise and non-invasive assessments of cardiovascular conditions, facilitating early diagnosis and better treatment planning (12). However, clinical studies involving stroke prevention and results reported more than 3 years after CAS are uncommon. Age, female sex, smoking, diabetes, dyslipidemia, hypertension, radiation to the head and neck, and hemodynamic conditions are all identified risk factors for in-stent restenosis (ISR); however, they vary according to the study (13). Most previous studies did not specify the processes or patient characteristics, imaging modalities used for ISR diagnosis, or universal ultrasonography criteria for ISR identification. Furthermore, few studies have used CDU in South Korea on factors related to restenosis following symptomatic and asymptomatic CAS (14). Therefore, this retrospective study aimed to assess the associated factors of significant restenosis after CAS with a median follow-up of 35.7 months.

## Materials and methods

### Participants

This retrospective study included patients who underwent CAS at Kyung Hee university hospital between January 2013 and December 2018. A total of 269 consecutive CAS was performed. Due to insufficient procedure date, pre- and post-CDU, and pre-digital subtraction angiography (DSA), 80 patients were excluded. We removed all personal identifiers from the dataset to ensure the privacy and confidentiality of the study participants. We included a total of 189 patients (age range: 49–90 years, mean age: 71 years).

We included the patients who underwent CAS for symptomatic or asymptomatic carotid stenosis. Symptomatic carotid stenosis was defined as stenosis of the internal carotid artery (ICA) causing ischemic stroke, transient ischemic attacks, or amaurosis fugax ipsilateral to the lesion in the past 6 months, with moderate ipsilateral carotid stenosis greater than 50% as determined by digital subtraction angiography (15). Asymptomatic carotid stenosis was defined as having at least 70% stenosis as identified by CDU, or at least 60% stenosis as determined by angiography (16).

Patients were excluded if DSA was not performed before CAS and if CDU was not performed at least once before and after CAS. Furthermore, patients with severe comorbidities (e.g., end-stage disease, severe renal failure, or liver failure) that could significantly impact study outcomes were also excluded.

**Figure 1:**
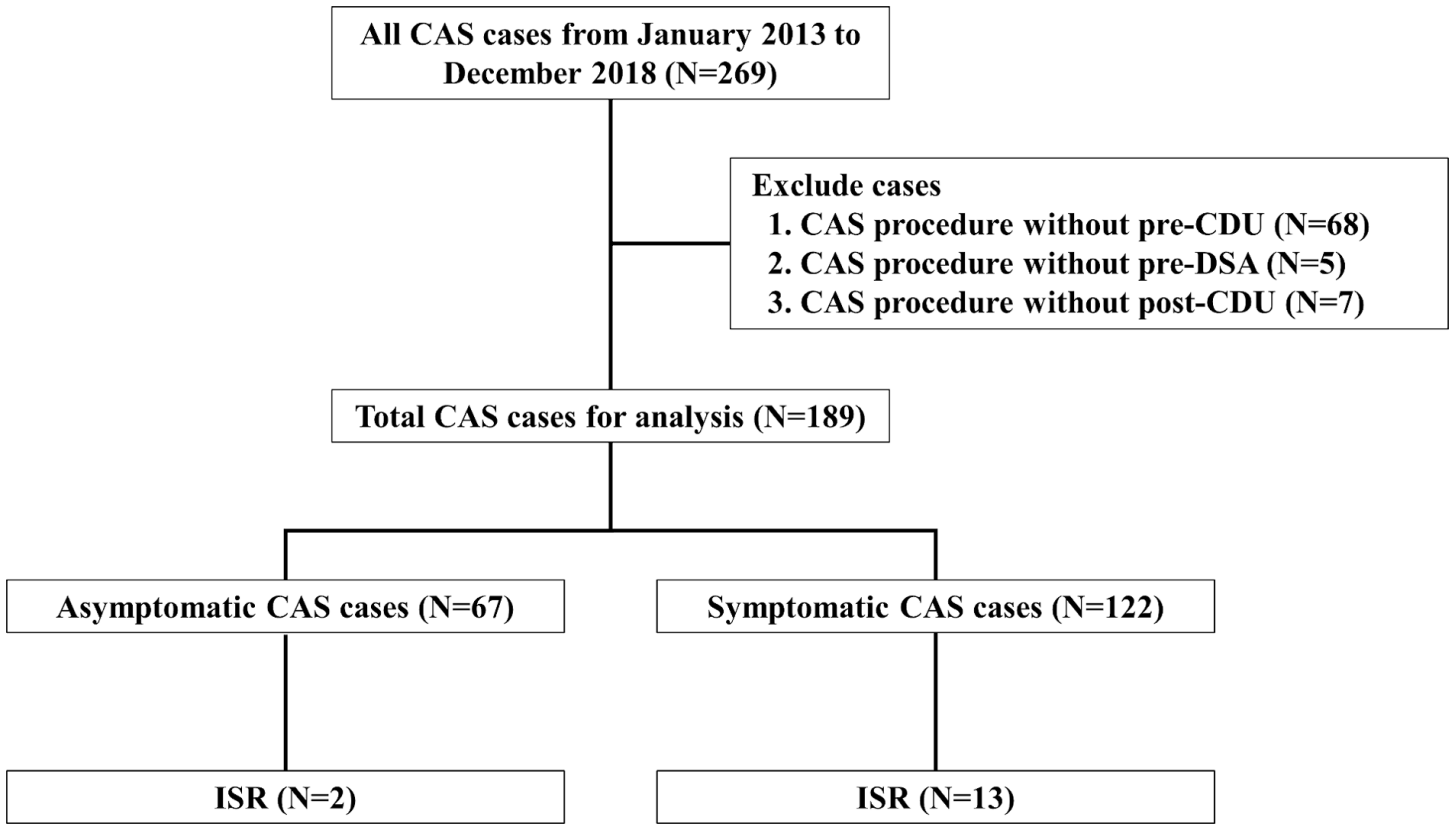
Flow diagram of patient enrollment Study flow chart depicting all patients enrolled in the study as well as the events precluding patients from this analysis. CAS, carotid artery angioplasty with stenting; CDU, carotid Doppler ultrasonography; DSA, digital subtraction angiography; ISR, in-stent restenosis.

Finally, 189 patients were enrolled for analysis; 122 had symptomatic stenosis, and 67 had asymptomatic stenosis (Figure 1). Missing values were addressed using the last observation carried forward (LOCF) method. For patients lost to follow-up, the last available data point was utilized. Additionally, regular clinic visits were scheduled at 6-month intervals. CDU assessments were conducted at each visit to monitor for in-stent restenosis and other relevant outcomes.

### Baseline risk factors and imaging parameters

Hospital records were used to gather information on patient demographics, CAS indications, and peri- and post-procedural results, including demographics (age, sex), medical history [diabetes mellitus, hypertension, dyslipidemia, prior stroke, chronic heart disease, history of active cancer, peripheral artery disease, atrial fibrillation, previous head and neck radiation therapy, current smoking (an adult who currently smokes cigarettes and has smoked 100 cigarettes during their lifetime), and moderate alcohol consumption (up to one drink per day for women and up to two for males is considered moderate drinking), imaging data, and procedural data].

DSA and CDU analyzed the most recent data before CAS. The pre- and peri-procedural image parameters included the location of the stenosis, presence of irregular or ulcerated plaque surfaces, plaque characteristics such as echogenic features, ICA peak systolic velocity (PSV), ICA end-diastolic velocity (EDV), ICA to common carotid artery (CCA) PSV ratio, residual stenosis rate (European carotid surgery trial (ECST) criteria), and presence of post-stent adjunctive balloon dilation. The North American symptomatic carotid endarterectomy trial (NASCET) criteria were used to estimate stenosis in the pre-procedure images.

### Carotid artery stent implantation and pre- and post-procedural management

At least 5 days before the stent placement, participants took dual antiplatelet agents such as aspirin (100 mg once daily), clopidogrel (75 mg once daily), or ticlopidine (250 mg twice daily). The CAS was performed under local anesthesia, and pre-stent balloon dilation was determined using DSA data on the degree of stenosis and the location of the stenotic artery. Neurointerventionists selected the stent in consideration of the length and composition of the lesion, vascular morphology, vessel diameter, and plaque characteristics. Only an inappropriate group of patients with more than 30% residual stenosis after stent expansion received adjunctive balloon dilation (17). All patients were administered dual antiplatelet medications (aspirin 100 mg/day, clopidogrel 75 mg/day, ticlopidine 500 mg/day, cilostazol 200 mg/day, or dipyridamole 400 mg/day) and moderate-to-high doses of statins according to the 2018 AHA guidelines for at least 3 months following CAS (18). Antiplatelet and statin were evaluated over the observation period, and statin intensity was classified as high, moderate, or low in accordance with the 2018 AHA guidelines. Low-density lipoprotein cholesterol (LDL) levels were assessed on the last day of the CDU examination.

### Carotid Doppler ultrasonography and in-stent restenosis

CDU evaluation was based on the guidelines for vascular ultrasonography published by the Korean Society of Neurosonology (19). Philips Affiniti 70 ultrasonography devices were adopted, and ultra-wideband probes of 5.0 to 12.0 MHz and micro-convex probes of 2.0 to 9.0 MHz were selected. The CDU was followed up after CAS, and the patients were recommended for follow-up at 6-month intervals after CAS. All ultrasonographic examinations were performed by a specialist at the cerebrovascular ultrasonography laboratory. The degree of stenosis and Doppler velocity were measured in the region with the greatest lumen reduction (Supplementary Figure 1). Although there is no accepted international standard for CDU-based ISR (20), we adopted modified criteria for diagnosis of ISR after CAS (if the ratio of PSV of the ICA to CCA is 3.4 or higher or PSV of ICA is 224 cm/s or higher in the CDU, stenosis degree is considered to be at least 50% or greater) (21). We investigated the factors that could increase the risk of ISR in the symptomatic and asymptomatic groups. All CDU examinations were performed by a specialist at the cerebrovascular ultrasonography laboratory.

### Statistical analysis

For continuous variables, results were presented as mean ± standard deviation (SD) or median (interquartile range), whereas for categorical variables, results were expressed as the number of participants (%). Fisher’s exact test was used to compare categorical variables, and an independent Student’s t-test was used to compare continuous variables. Given the small sample size, variables with *p* < 0.2 from the univariate logistic regression analysis were included in the multivariable logistic regression model using the backward elimination technique to evaluate independent risk factors for ISR (22). We also incorporated variables that are well-established in the literature as potential risk factors for ISR, even if they did not meet the statistical threshold in our univariate analysis. Prior to inclusion in the multivariable logistic regression analysis, multicollinearity was assessed using the variance inflation factor (VIF), and variables with high VIFs were excluded to ensure model stability and interpretability. The odds ratios (OR) and 95% confidence intervals (CI) were obtained. Kaplan–Meier analysis of the symptomatic and asymptomatic CAS groups was used to assess cumulative ISR-free time. All tests were conducted on both sides, and *p* < 0.05 was considered statistically significant. Statistical Package for the Social Sciences software (version 23.0; IBM Corp., Armonk) was used for the statistical analysis.

## Results

### Patients’ characteristics

A total of 67 asymptomatic patients underwent CAS, with a mean age of 70.4 ± 7.9 years (mean ± standard deviation), and 59 (88.1%) were males. There were 30 (44.8%) patients with diabetes, 58 (86.6%) with hypertension, 45 (67.2%) with dyslipidemia, and 15 (22.4%) were current smokers, with mean stenosis of 80.0% ± 9.5%, mean ICA PSV of 315.9 ± 153.2 cm/s, and ICA/CCA PSV ratio of 4.5% ± 2.5 (Tables 1, 2).

**Table 1:** Baseline characteristics of all patients undergoing carotid artery stenting.

Variable	Overall (*n* = 189)	Asymptomatic CAS (*n* = 67)	Symptomatic CAS (*n* = 122)	*P*	ISR(−) group (*n* = 174)	ISR(+) group (*n* = 15)	*P*
Age, years	70.4 ± 7.7	70.4 ± 7.9	70.4 ± 7.6	0.971	70.6 ± 7.7	68.3 ± 8.3	0.287
Male sex	166 (87.8)	59 (88.1)	107 (87.7)	0.943	152 (87.4)	14 (93.3)	0.497
**Vascular risk factor**							
Diabetes	86 (45.5)	30 (44.8)	56 (45.9)	0.882	77 (44.3)	9 (60.0)	0.240
Hypertension	162 (85.7)	58 (86.6)	104 (85.2)	0.804	149 (85.6)	13 (86.7)	0.913
Dyslipidemia	139 (73.5)	45 (67.2)	94 (77.0)	0.141	127 (73.0)	12 (80.0)	0.555
Previous stroke	98 (51.9)	30 (44.8)	68 (55.7)	0.149	92 (52.9)	6 (40.0)	0.338
Chronic heart disease	47 (24.9)	22 (32.8)	35 (28.7)	0.552	51 (29.3)	6 (40.0)	0.387
Cancer	18 (9.5)	9 (13.4)	9 (7.4)	0.175	18 (10.3)	0 (0.0)	0.190
Peripheral artery disease	4 (2.1)	2 (3.0)	2 (1.6)	0.539	4 (2.3)	0 (0.0)	0.553
Atrial fibrillation	10 (5.3)	1 (1.5)	9 (7.4)	0.084	9 (5.2)	1 (6.7)	0.804
HNRT	7 (3.7)	**5 (7.5)**	**2 (1.6)**	**0.043**	7 (4.0)	0 (0.0)	0.429
Current smoking	47 (24.9)	15 (22.4)	32 (26.2)	0.559	**40 (23.0)**	**7 (46.7)**	**0.042**
Alcohol	70 (37.0)	21 (31.3)	49 (40.2)	0.230	61 (35.1)	9 (60.0)	0.055
Symptomatic CAS	122 (64.6)	0 (0.0)	122 (100.0)	-	109 (62.6)	13 (86.7)	0.062
ISR	15 (7.9)	2 (3.0)	13 (10.7)	0.062	0 (0.0)	15 (100.0)	-
Follow-up period, months (median, IQR)	35.7 (19.5 to 70.0)	47.0 (21.0 to 78.0)	34.0 (19.0 to 69.3)	0.204	**41.0 (20.8 to 76.5)**	**24.0 (12.0 to 33.0)**	**<0.001**
**Laboratory parameters**							
White blood cell count, cells/mL	7.0 ± 2.0	6.9 ± 1.9	7.1 ± 2.0	0.404	7.0 ± 1.9	7.2 ± 1.8	0.651
Hemoglobin, g/dL	13.4 ± 1.9	**12.9 ± 2.2**	**13.6 ± 1.7**	**0.024**	13.3 ± 1.9	13.8 ± 2.3	0.301
Platelet count, cells/mL	233.0 ± 62.0	241.1 ± 56.8	228.5 ± 64.4	0.181	233.2 ± 60.6	230.1 ± 78.3	0.850
Creatinine, mg/dL	1.0 ± 0.3	1.0 ± 0.3	1.0 ± 0.4	0.986	1.0 ± 0.3	1.0 ± 0.5	0.485
Total cholesterol, mg/dL	152.3 ± 48.4	144.1 ± 48.6	156.8 ± 47.9	0.084	153.4 ± 48.9	139.1 ± 42.0	0.272
TG, mg/dL	127.9 ± 75.3	124.2 ± 66.8	129.9 ± 79.9	0.622	127.3 ± 75.1	135.1 ± 81.1	0.699
HDL-C, mg/dL	42.7 ± 12.9	41.8 ± 10.9	43.2 ± 13.9	0.473	42.9 ± 12.9	40.8 ± 13.6	0.554
Baseline LDL-C, mg/dL	93.4 ± 38.6	88.6 ± 37.6	96.0 ± 39.1	0.211	94.5 ± 39.1	80.8 ± 31.2	0.190
HbA1C, %	6.5 ± 1.3	6.4 ± 1.1	6.5 ± 1.3	0.740	6.5 ± 1.3	6.2 ± 0.6	0.482

CAS, carotid artery angioplasty with stenting; HbA1C, glycated hemoglobin; HDL-C, high-density lipoprotein cholesterol; HNRT, head and neck radiation therapy; LDL-C, low-density lipoprotein cholesterol; IQR, interquartile range; ISR, in-stent restenosis; TG, triglyceride. The data are mean ± standard deviation or numbers of patients (%). *p*-value for Fisher’s exact test for categorical variables and *t*-test for continuous variables. The values with bold type represent statistically significant results with a *p*-value < 0.05.

**Table 2:** Baseline imaging characteristics of the total number of patients.

Variable	Overall (*n* = 189)	Asymptomatic CAS (*n* = 67)	Symptomatic CAS (*n* = 122)	*P*	ISR(−) group (*n* = 174)	ISR(+) group (*n* = 15)	*P*
**DSA**							
Stenosis site, left	104 (55.0)	41 (61.2)	63 (51.6)	0.207	97 (55.7)	7 (46.7)	0.498
Pre-procedure degree of stenosis (NASCET), %	79.6 ± 10.3	80.0 ± 9.5	79.4 ± 10.7	0.703	79.5 ± 10.5	80.9 ± 7.9	0.612
Stenosis location				0.276			0.313
Common carotid artery	2 (1.1)	5 (7.5)	5 (4.1)		8 (4.6)	2 (13.3)	
Bifurcation	56 (29.6)	0 (0.0)	3 (2.5)		3 (1.7)	0 (0.0)	
Internal carotid artery	131 (69.3)	62 (92.5)	114 (93.4)		163 (93.7)	13 (86.7)	
Irregular or ulcerated plaque surface^†^	153 (81.0)	58 (86.6)	95 (77.9)	0.145	142 (81.6)	11 (73.3)	0.434
Calcification	6 (3.2)	4 (6.0)	31 (25.4)	0.104	6 (3.4)	0 (0.0)	0.465
**CDU**							
ICA PSV, cm/s	333.6 ± 165.8	315.9 ± 153.2	343.4 ± 172.1	0.276	**326.0 ± 165.2**	**422.5 ± 151.3**	**0.030**
ICA EDV, cm/s	128.9 ± 89.7	121.2 ± 78.2	133.1 ± 95.4	0.385	126.0 ± 90.0	162.6 ± 83.9	0.129
CCA PSV, cm/s	80.7 ± 56.6	80.0 ± 9.5	81.6 ± 64.5	0.769	81.5 ± 58.7	71.5 ± 21.9	0.514
CCA EDV, cm/s	20.6 ± 13.7	21.0 ± 12.0	20.3 ± 14.6	0.761	20.8 ± 14.1	18.0 ± 8.2	0.457
ICA/CCA PSV ratio	5.2 ± 3.6	**4.5 ± 2.5**	**5.6 ± 4.0**	**0.021**	5.0 ± 3.4	7.0 ± 3.4	0.457
Echolucent	49 (25.9)	18 (26.9)	31 (25.4)	0.827	46 (26.4)	3 (20.0)	0.585
**Procedural factors**							
Post-stent adjunctive balloon dilation	23 (12.2)	**14 (20.9)**	**9 (7.4)**	**0.007**	23 (13.2)	0 (0.0)	0.133
Post-procedure degree of stenosis (ECST), %	20.7 ± 12.2	19.5 ± 12.5	21.3 ± 12.1	0.333	20.5 ± 12.5	23.1 ± 8.9	0.434

CAS, carotid artery angioplasty with stenting; CCA, common carotid artery; CDU, carotid Doppler ultrasonography; DSA, digital subtraction angiography; ECST, European carotid surgery trial; EDV, end-diastolic velocity; ICA, internal carotid artery; ISR, in-stent restenosis; NASCET, North American symptomatic carotid endarterectomy trial; PSV, peak systolic velocity. ^†^Plaque classification based on the 1994 American Heart Association standard criteria. The data are mean ± standard deviation or numbers of patients (%). *P*-value for Fisher’s exact test for categorical variables and Student’s t-test for continuous variables. The values with bold type represent statistically significant results with a *p*-value < 0.05.

On the other hand, 122 symptomatic patients underwent CAS, with a mean age of 70.4 ± 7.6 years, and 107 (87.7%) were males. There were 56 (45.9%) patients with diabetes, 104 (85.2%) with hypertension, 94 (77.0%) with dyslipidemia, and 32 (26.2%) were current smokers, with mean stenosis of 79.4 ± 10.7%, 9 (7.4%) with post-stent adjunctive balloon dilation, mean ICA PSV of 343.4 ± 172.1 cm/s, and ICA/CCA PSV ratio of 5.6 ± 4.0 (Tables 1, 2).

### In-stent restenosis

Patients were followed up by ultrasonography for a median of 35.7 months [interquartile range (IQR), 19.5 to 70.0], and 15 (7.9%) had ISR. The rates of ISR were not significantly different in patients with asymptomatic and symptomatic CAS groups (3.0% vs. 10.7%, *p* = 0.062). In the asymptomatic CAS group, the rates of head and neck radiation therapy (7.5% vs. 1.6%, *p* = 0.043) were significantly higher, and baseline hemoglobin (12.9 ± 2.2 vs. 13.6 ± 1.7, *p* = 0.024) was significantly lower. In the ISR group, the rates of current smokers (23.0% vs. 46.7%, *p* = 0.042) and ICA PSV at baseline were significantly increased (326.0 ± 165.2 cm/s vs. 422.5 ± 151.3 cm/s, *p* = 0.030). Furthermore, the rates of post-stent adjunctive balloon dilation were significantly lower in the symptomatic CAS group (20.9% vs. 7.4%, *p* = 0.007; Tables 1, 2).

At least one antiplatelet medication was maintained by the patient 3 months after the procedure, and the ISR group had a high rate of dual antiplatelet medication use (64.4% vs. 80.0%, *p* = 0.221). The symptomatic CAS group had administered more high-intensity statins (22.4% vs. 41.8%, *p* = 0.019), while the final LDL level of the ISR group was significantly lower (74.5 ± 25.6 vs. 60.1 ± 19.4, *p* = 0.034; Table 3).

**Table 3:** Comparison of maintenance therapy for patients with or without restenosis of all patients undergoing CAS.

Variable	Overall (*n* = 189)	Asymptomatic CAS (*n* = 67)	Symptomatic CAS (*n* = 122)	*P*	ISR (−) group (*n* = 174)	ISR (+) group (*n* = 15)	*P*
Baseline LDL-C, mg/dL	93.4 ± 38.6	88.6 ± 37.6	96.0 ± 39.1	0.211	94.5 ± 39.1	80.8 ± 31.2	0.190
Final LDL-C, mg/dL	73.4 ± 25.4	76.4 ± 23.1	71.7 ± 26.5	0.227	**74.5 ± 25.6**	**60.1 ± 19.4**	**0.034**
Antiplatelet treatment				0.531			0.221
Mono therapy	65 (34.4)	25 (37.3)	40 (32.8)		62 (35.6)	3 (20.2)	
Dual therapy	124 (65.6)	42 (62.7)	82 (67.2)		112 (64.4)	12 (80.0)	
Type of statins				0.365			0.543
Atorvastatin	146 (77.2)	51 (76.1)	95 (77.9)		133 (76.4)	13 (86.4)	
Rosuvastatin	32 (16.9)	10 (14.9)	22 (18.0)		31 (17.8)	1 (6.7)	
Others	11 (5.8)	6 (9.0)	5 (4.1)		10 (5.7)	1 (6.7)	
Lipid lowering treatment^†^				**0.019**			0.264
Low intensity statin	5 (2.6)	**3 (4.5)**	**2 (1.6)**		5 (2.9)	0 (0.0)	
Moderate intensity statin	118 (62.4)	**49 (73.1)**	**69 (56.6)**		111 (63.8)	7 (46.7)	
High intensity statin	66 (34.9)	**15 (22.4)**	**51 (41.8)**		58 (33.3)	8 (53.3)	

CAS, carotid artery angioplasty with stenting; LDL-C, low-density lipoprotein cholesterol; ISR, in-stent restenosis; TG, triglyceride. ^†^The intensity of statin therapy was categorized according to the 2018 American Heart Association guidelines. The data are mean ± standard deviation or numbers of patients (%). *P*-value for Fisher’s exact test for categorical variables and Student’s t-test for continuous variables. The values with bold type represent statistically significant results with a *p*-value < 0.05.

In the overall CAS group, current smoking (OR, 2.931; 95% CI, 1.001 to 8.581) and elevated ICA PSV at baseline (OR, 1.004; 95% CI, 1.001 to 1.007) were associated with ISR using univariate logistic regression analysis (Supplementary Table 1). In multivariable logistic regression analysis, current smoking (adjusted odds ratio (aOR), 3.425; 95% CI, 1.086 to 10.801) and elevated ICA PSV at baseline (aOR, 1.004; 95% CI, 1.001 to 1.007) were independently associated with the ISR (Table 4).

**Table 4:** Multivariable logistic regression analysis of clinical variables for carotid restenosis after CAS.

Variable	^†^Adjusted odds ratio (95% CI)	*P*
ICA PSV, cm/s	**1.004 (1.001 to 1.007)**	**0.037**
Current smoking	**3.425 (1.086 to 10.801)**	**0.036**
Baseline LDL-C, mg/dL	0.984 (0.968 to 1.001)	0.066
Symptomatic CAS	4.453 (0.913 to 21.720)	0.065

CAS, carotid artery angioplasty with stenting; CI, confidence interval; ICA, internal carotid artery; LDL-C, low-density lipoprotein cholesterol; PSV, peak systolic velocity. ^†^Adjusted for age, sex, current smoking, alcohol, LDL-C, symptomatic CAS, and ICA PSV. The values with bold type represent statistically significant results with a *p*-value < 0.05.

In the symptomatic CAS group, elevated ICA PSV at baseline (OR, 1.004; 95% CI, 1.001 to 1.007) was associated with ISR using univariate logistic regression analysis (Supplementary Table 1). In multivariable logistic regression analysis, alcohol (aOR, 4.239; 95% CI, 1.090 to 16.484) was independently associated with the ISR (Supplementary Table 2).

**Figure 2:**
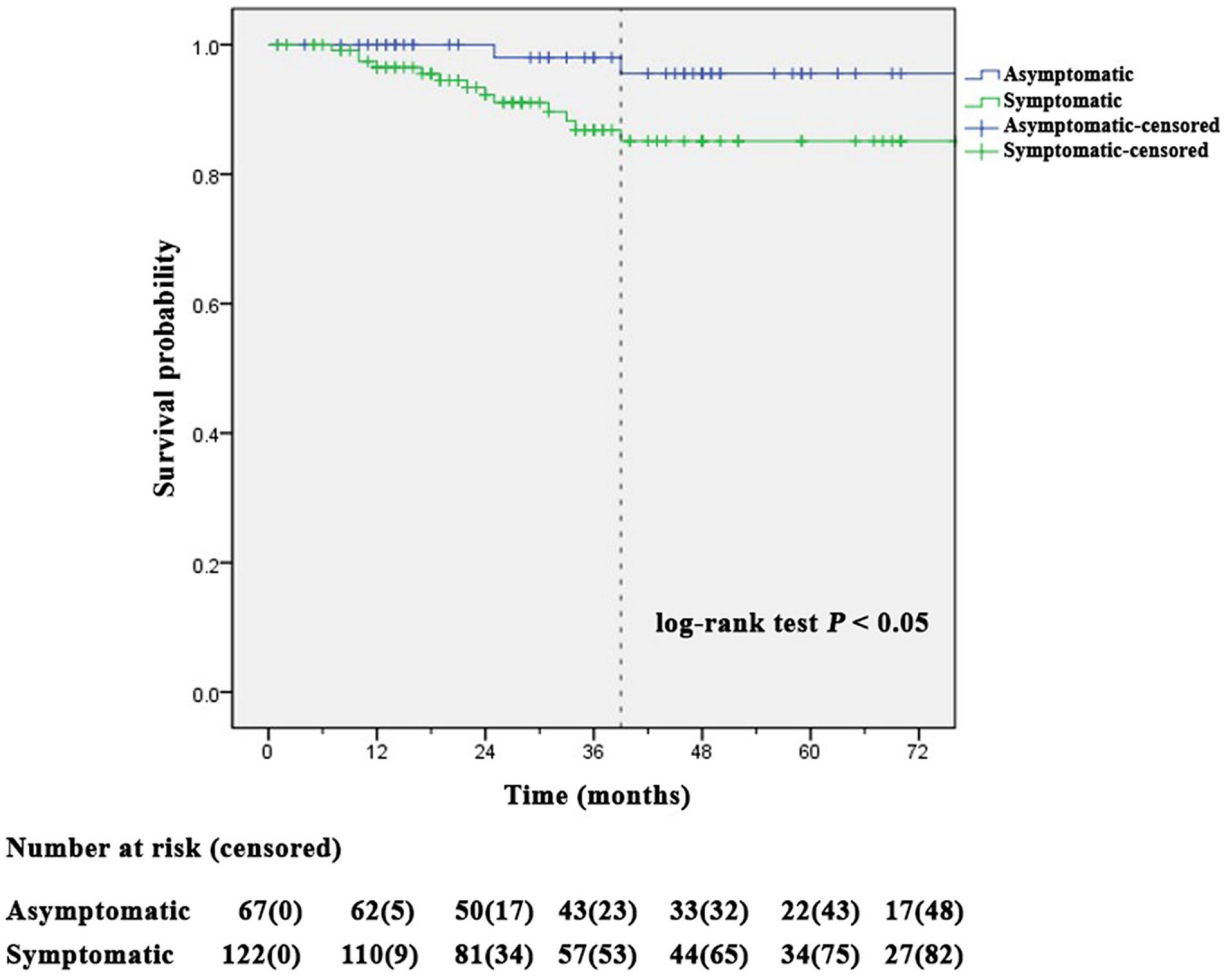
Kaplan–Meier analysis showed a significant difference in stent restenosis-free time between symptomatic and asymptomatic carotid artery angioplasty with stenting groups (log-rank test *p* = 0.043).

Kaplan–Meier analysis showed ISR-free time between the symptomatic and asymptomatic CAS groups (Figure 2). In patients with asymptomatic CAS, the mean time of ISR-free time was 135.2 ± 3.3 months (mean ± SD), and the longest time to ISR noted was 39 months. In patients with symptomatic CAS, the mean time of ISR-free was 127.8 ± 4.7 months, and the longest time to ISR was 39 months. Compared with the symptomatic CAS group, ISR-free was better in the asymptomatic CAS group (*p* = 0.043).

## Discussion

### Findings of our study

This study demonstrated that ISR was significantly associated with current smoking and elevated ICA PSV in the CAS group. Additionally, alcohol consumption was an independent risk factor for ISR in the symptomatic CAS group. According to the Kaplan–Meier analysis, ISR occurred in all CAS groups within 39 months of follow-up, and the ISR-free time was longer in the asymptomatic CAS group than that in the symptomatic CAS group. Even after 3 months, the patients continued to take antiplatelet drugs at least once, and the ISR group had a higher rate of dual antiplatelet medication use. The final LDL level in the ISR group was significantly lower than that in the symptomatic CAS group, which received high-intensity statins.

### Plausible mechanism

Since a few decades ago, there has been an interest in the mechanism of extracranial carotid atherosclerosis (ECAS) incidence in smokers, which is not well known. Many studies have suggested that smoking is a major risk factor for carotid atherosclerosis (23). This research has consistently demonstrated that smoking increases the risk of ISR. It is estimated that smoking causes oxidative stress, vascular inflammation, platelet coagulation, vascular dysfunction, and impaired serum lipid profiles and has a detrimental effect on the cardiovascular system (24).

Previous studies have shown that ICA PSV is better associated with lumen stenosis and is less affected by distal ICA occlusive diseases, such as moyamoya disease (25). ICA PSV measurements show changes in physiological flow with increasing carotid stenosis levels (26). Endothelial cells surrounding artery walls are sensitive to the mechanical pressures generated by blood flow (27). It is possible that the mechanical sensitivity of the endothelial cell has increased after being exposed to elevated ICA PSV over a prolonged period of time.

The effects of alcohol on carotid atherosclerosis have been previously studied, but the outcomes of these studies have been inconsistent (28). An earlier study found that alcohol is substantially correlated with carotid intima-media thickness in young adults between the ages of 24 and 39 years, even after considering age, sex, and cardiovascular risk factors (29). This finding suggests that alcohol may promote atherosclerosis. It is assumed that because there were more alcohol drinkers in the symptomatic CAS group, alcohol may have had a more significant impact on ISR.

Statins have anti-inflammatory effects that stabilize carotid artery damage and may even slow or stop plaque growth over time (30). Interestingly, after the procedure, high-intensity statins were provided to the high-risk group to lower LDL levels, but the final LDL level was significantly lower in the ISR group. This suggests that the causes of ISR are complex.

### Comparison with previous studies and clinical implications

Recent investigations have shown that CAS is a successful treatment option for patients with carotid atherosclerotic stenosis (31). In the carotid revascularization endarterectomy vs. stenting trial, the incidence of an ipsilateral stroke was four times higher in participants with restenosis or occlusion within 2 years than in those without restenosis or occlusion (32). A critical problem with CAS is the formation of in-stent neointimal proliferation, which may result in restenosis of the stented vessel (33). ISR was likely to occur after more than 12 months (34). Rates of ISR in other studies with extensive follow-up ranged from 1.7% to 21%, and the incidence of ISR was 6% after 2 years (32). Our study found that the last ISR occurred at 39 months, and the prevalence of ISR (>50%) over the entire period was 7.9% in the overall groups, 3.0% in the asymptomatic group, and 10.7% in the symptomatic group, which was similar to the those of a previous study. The Society for Vascular Surgery recommends that CDU be performed at baseline, every 6 months for 2 years, and annually thereafter if the stent insertion site does not demonstrate stenosis (35). Considering the occurrence of ISR for up to 39 months, we propose to perform regular CDU for the first 3 years.

The ISR has been investigated in previous studies. However, most studies have been conducted in America or Europe (36, 37). In previous research, age, female sex, diabetes, angina, a higher degree of stenosis in the opposite carotid artery, hypertension, dyslipidemia, and smoking were considered independent predictors of ISR (38). Except for current smoking, elevated ICA PSV, and alcohol consumption, our study showed no other parameters to be substantially correlated with ISR. According to the Korea National Health and Nutrition Examination Survey for 2008–2017, decreased sodium intake and metabolic syndrome prevalence were stabilized (39). Therefore, it is possible that better control of diseases, including diabetes, hypertension, dyslipidemia, and smoking, has resulted in a decrease in ISR.

The cumulative 5-year risk of ISR (>50%) was 40.7% based on the second analysis of the international carotid stenting study (38). In our study, Kaplan–Meier analysis showed that the cumulative ISR-free over the median follow-up period of 35.7 months. Was 95.6% in the asymptomatic CAS group and 85.1% in the symptomatic CAS group. The reason the cumulative ISR-free over the median follow-up period of 35.7 months was higher than that in previous studies may be that our stroke center was a certified training center, and interventionists had more than 10 years of experience. The ACC/AHA and the European Society for Vascular Surgery guidelines suggest that patients undergo carotid artery stenting performed in a hospital with a periprocedural complication rate of less than 6% (40). The incidence of complications during the procedure in this study was 2.1%, and the ability of the interventionists likely had an impact on the reduction in ISR.

Most prior studies have demonstrated that antiplatelet therapy does not influence ISR, a finding that aligns with the results of our investigation (41). Conversely, there are studies indicating that statin use does affect ISR, which contrasts with our findings (42). This discrepancy is likely attributable to variations in baseline demographics, such as initial LDL levels, symptom status, and ICA PSV levels.

The findings of our study have several important practical implications for the management of carotid artery stenosis. Our information can be used to enhance screening and monitoring protocols for patients undergoing CAS, allowing for more personalized follow-up schedules and interventions. Furthermore, this could improve patient outcomes by selecting those who are less likely to experience restenosis and by considering alternative treatments for higher-risk individuals.

### Limitations

This study has some limitations. First, it used a small sample size and a non-randomized design in a single center. Therefore, this result is vulnerable to selection bias, provides restricted power for logistic regression analysis, and cannot be generalized to the global population. Second, the interval between the two rounds of CDU was irregular. This may have underestimated the prevalence of ISR. Third, follow-up imaging, including magnetic resonance angiography, computed tomography angiography, and DSA after CAS, were not included in this study, as only CDU was followed. It is impossible to rule out false-positive results when predicting ISR through the PSV of CDU because the opposite carotid stenosis or occlusion may increase the blood flow velocity in-stent lesions. Fourth, the presence of residual confounding factors could not be completely eliminated, even after adjusting for primary risk factors and they may still have clinical relevance and warrant further investigation in future studies with larger sample sizes. Fifth, the rate of ISR may have differed because there are no universally accepted ultrasonography criteria for detecting it. The outcome may change depending on the adopted ISR criteria. Finally, the duration of smoking and drinking was not considered. Previous research has shown that alcohol use and atherosclerosis have a J-shaped relationship and that smoking increases the risk of developing ECAS (43). Additional research focusing on the duration of drinking and smoking is required.

## Conclusion

Independent risk factors for ISR in the CAS group included elevated ICA PSV and current smoking at baseline. In the symptomatic CAS group, alcohol was independently associated with the ISR. ISR is often present within 39 months after the CAS procedure based on our median follow-up of 35.7 months. However, we recognize that a longer follow-up period could provide additional insights into the timing and frequency of ISR development. Future studies with extended follow-up are necessary to fully understand the long-term outcomes of CAS.

## Data availability statement

The original contributions presented in the study are included in the article/Supplementary material, further inquiries can be directed to the corresponding author.

## Ethics statement

The studies involving humans were approved by Institutional Review Board (IRB) of Kyung Hee University Hospital (KMC IRB 2009-12-301). The studies were conducted in accordance with the local legislation and institutional requirements. Written informed consent for participation was not required from the participants or the participants’ legal guardians/next of kin because Informed consent was waived owing to the retrospective nature of the study.

## Author contributions

SP: Conceptualization, Data curation, Formal analysis, Investigation, Methodology, Software, Validation, Writing – original draft, Writing – review & editing. BK: Conceptualization, Writing – review & editing. H-YC: Conceptualization, Writing – review & editing. D-IC: Conceptualization, Writing – review & editing. HW: Conceptualization, Formal analysis, Investigation, Methodology, Supervision, Writing – original draft, Writing – review & editing, Data curation. SH: Conceptualization, Formal analysis, Investigation, Supervision, Writing – original draft, Writing – review & editing, Funding acquisition, Methodology, Project administration, Validation.
