# Supplementary material for: Risk factors of in-stent restenosis after carotid angioplasty and stenting: long-term follow-up study
Source: Front Neurol. 2024 Aug 8;15:1411045. doi: 10.3389/fneur.2024.1411045 (PMC11340531; doi:10.3389/fneur.2024.1411045)
Supplement: Supplementary file 1 [file Data_Sheet_1.docx]

**Figure legends**

**Supplemental Figure 1.** Typical carotid Doppler spectrum of the internal carotid artery

The Doppler velocity is measured at the region with the greatest lumen reduction.

**Supplemental Table 1.** Univariate logistic regression analysis of factors related to restenosis after CAS

| **Variable** | **Overall (n = 189)** | | **Symptomatic CAS (n = 122)** | |
| --- | --- | --- | --- | --- |
|  | **OR (95% CI)** | ***P*** | **OR (95% CI)** | ***P*** |
| **Age, years** | 0.965 (0.903 to 1.031) | 0.287 | 0.961 (0.894 to 1.032) | 0.273 |
| **Male sex** | 2.026 (0.254 to 16.178) | 0.505 | 1.768 (0.213 to 14.671) | 0.597 |
| **Vascular risk factor** |  |  |  |  |
| Diabetes mellitus | 1.890 (0.645 to 5.539) | 0.246 | 2.968 (0.861 to 10.229) | 0.085 |
| Hypertension | 1.091 (0.232 to 5.127) | 0.913 | 0.946 (0.192 to 4.674) | 0.946 |
| Dyslipidemia | 1.480 (0.400 to 5.479) | 0.557 | 1.723 (0.359 to 8.279) | 0.497 |
| Previous stroke | 0.594 (0.203 to 1.741) | 0.343 | 0.456 (0.140 to 1.485) | 0.193 |
| Chronic heart disease | 1.608 (0.544 to 4.750) | 0.390 | 2.365 (0.734 to 7.620) | 0.149 |
| Atrial fibrillation | 1.130 (0.155 to 11.095) | 0.805 | 1.052 (0.121 to 9.153) | 0.963 |
| Current smoking status | **2.931 (1.001 to 8.581)** | **0.049** | 1.898 (0.572 to 6.296) | 0.295 |
| Alcohol | 2.779 (0.945 to 8.173) | 0.063 | 2.654 (0.813 to 8.659) | 0.106 |
| **Laboratory parameters** |  |  |  |  |
| Hemoglobin, g/dL | 1.191 (0.861 to 1.646) | 0.291 | 1.005 (0.722 to 1.400) | 0.976 |
| Platelet count, cells/mL | 0.999 (0.991 to 1.008) | 0.849 | 0.998 (0.989 to 1.007) | 0.726 |
| Creatinine, mg/dL | 1.581 (0.437 to 5.723) | 0.485 | 1.744 (0.494 to 6.160) | 0.388 |
| Total cholesterol | 0.993 (0.981 to 1.005) | 0.267 | 0.990 (0.977 to 1.004) | 0.155 |
| TG, mg/dL | 1.001 (0.995 to 1.008) | 0.698 | 1.001 (0.995 to 1.008) | 0.669 |
| HDL-C, mg/dL | 0.986 (0.941 to 1.003) | 0.550 | 0.984 (0.937 to 1.033) | 0.515 |
| Baseline LDL-C, mg/dL | 0.989 (0.972 to 1.005) | 0.188 | 0.986 (0.968 to 1.004) | 0.125 |
| HbA1C, % | 0.827 (0.488 to 1.403) | 0.482 | 0.889 (0.537 to 1.471) | 0.646 |
| **Symptomatic CAS** | 3.876 (0.848 to 17.724) | 0.081 | - | - |
| **DSA** |  |  |  |  |
| Stenosis site, left | 0.695 (0.241 to 2.000) | 0.499 | 0.550 (0.169 to 1.787) | 0.320 |
| Pre-procedure degree of stenosis (NASCET), % | 1.014 (0.961 to 1.070) | 0.610 | 1.020 (0.963 to 1.080) | 0.504 |
| Irregular or ulcerated plaque surface * | 0.620 (0.185 to 2.072) | 0.437 | 0.602 (0.170 to 2.131) | 0.431 |
| **CDU** |  |  |  |  |
| ICA PSV, cm/s | **1.004 (1.001 to 1.007)** | **0.035** | **1.004 (1.001 to 1.006)** | **0.034** |
| ICA EDV, cm/s | 1.004 (0.999 to 1.009) | 0.134 | 1.004 (0.999 to 1.010) | 0.122 |
| CCA PSV, cm/s | 0.995 (0.978 to 1.011) | 0.520 | 0.996 (0.982 to 1.010) | 0.590 |
| CCA EDV, cm/s | 0.978 (0.923 to 1.036) | 0.447 | 0.980 (0.924 to 1.039) | 0.980 |
| Echolucent | 0.696 (0.188 to 2.576) | 0.587 | 0.868 (0.223 to 3.381) | 0.838 |
| **Procedural factors** |  |  |  |  |
| Post-procedure degree of stenosis (ECST), % | 1.017 (0.975 to 1.060) | 0.433 | 1.019 (0.974 to 1.066) | 0.413 |
| Final LDL-C, mg/dL | **0.967 (0.938 to 0.997)** | **0.031** | **0.968 (0.937 to 0.999)** | **0.045** |
| **Antiplatelet treatment** |  | 0.232 |  | 0.174 |
| Mono therapy | reference |  | reference |  |
| Dual therapy | 2.214 (0.602 to 8.146) |  | 2.944 (0.620 to 13.970) |  |
| **Type of statins** |  | 0.574 |  | 0.524 |
| Atorvastatin | reference |  | reference |  |
| Rosuvastatin | 0.330 (0.042 to 2.618) | 0.294 | 0.364 (0.044 to 2.976) | 0.346 |
| Others | 1.023 (0.121 to 8.636) | 0.983 | 1.909 (0.195 to 18.658) | 0.578 |
| **Lipid lowering treatment** **^†^** |  | 0.353 |  | 0.355 |
| Moderate intensity statin | 0.457 (0.158 to 1.324) | 0.149 | 0.420 (0.129 to 1.370) | 0.150 |
| High intensity statin | reference |  | reference |  |

CAS, carotid artery angioplasty with stenting; CCA, common carotid artery; CDU, carotid Doppler ultrasonography; CI, confidence interval; ECST, European carotid surgery trial; EDV, end-diastolic velocity; DSA, digital subtraction angiography; HbA1C, glycated hemoglobin; HDL-C, high-density lipoprotein cholesterol; ICA, internal carotid artery; ISR, in-stent restenosis; LDL-C, low-density lipoprotein cholesterol; NASCET, North American symptomatic carotid endarterectomy trial; OR, odds ratio; PSV, peak systolic velocity; TG, triglyceride.

* Plaque classification based on the 1994 American Heart Association standard criteria.

**^†^** The intensity of statin therapy is categorized according to the 2018 American Heart Association guidelines.

Numbers in bold indicate a

**Supplemental Table 2.** Multivariate logistic regression analysis of clinical variables for carotid restenosis after symptomatic CAS

| Variable | *odds ratio (95% CI) | *P* |
| --- | --- | --- |
| ICA PSV, cm/s | 1.004 (1.000 to 1.008) | 0.056 |
| Diabetes mellitus | 3.953 (1.000 to 15.626) | 0.050 |
| Alcohol | **4.239 (1.090 to 16.484)** | **0.037** |
| Baseline LDL-C, mg/dL | 0.982 (0.963 to 1.001) | 0.067 |

CAS, carotid artery angioplasty with stenting; CI, confidence interval; ICA, internal carotid artery; LDL-C, low-density lipoprotein cholesterol; PSV, peak systolic velocity.

*Adjusted for age, sex, diabetes mellitus, previous stroke, chronic heart disease, alcohol, total cholesterol, LDL-C, and ICA PSV.
