# Supplementary figures and images for: Risk factors of in-stent restenosis after carotid angioplasty and stenting: long-term follow-up study
Source: Front Neurol. 2024 Aug 8;15:1411045. doi: 10.3389/fneur.2024.1411045 (PMC11340531; doi:10.3389/fneur.2024.1411045)

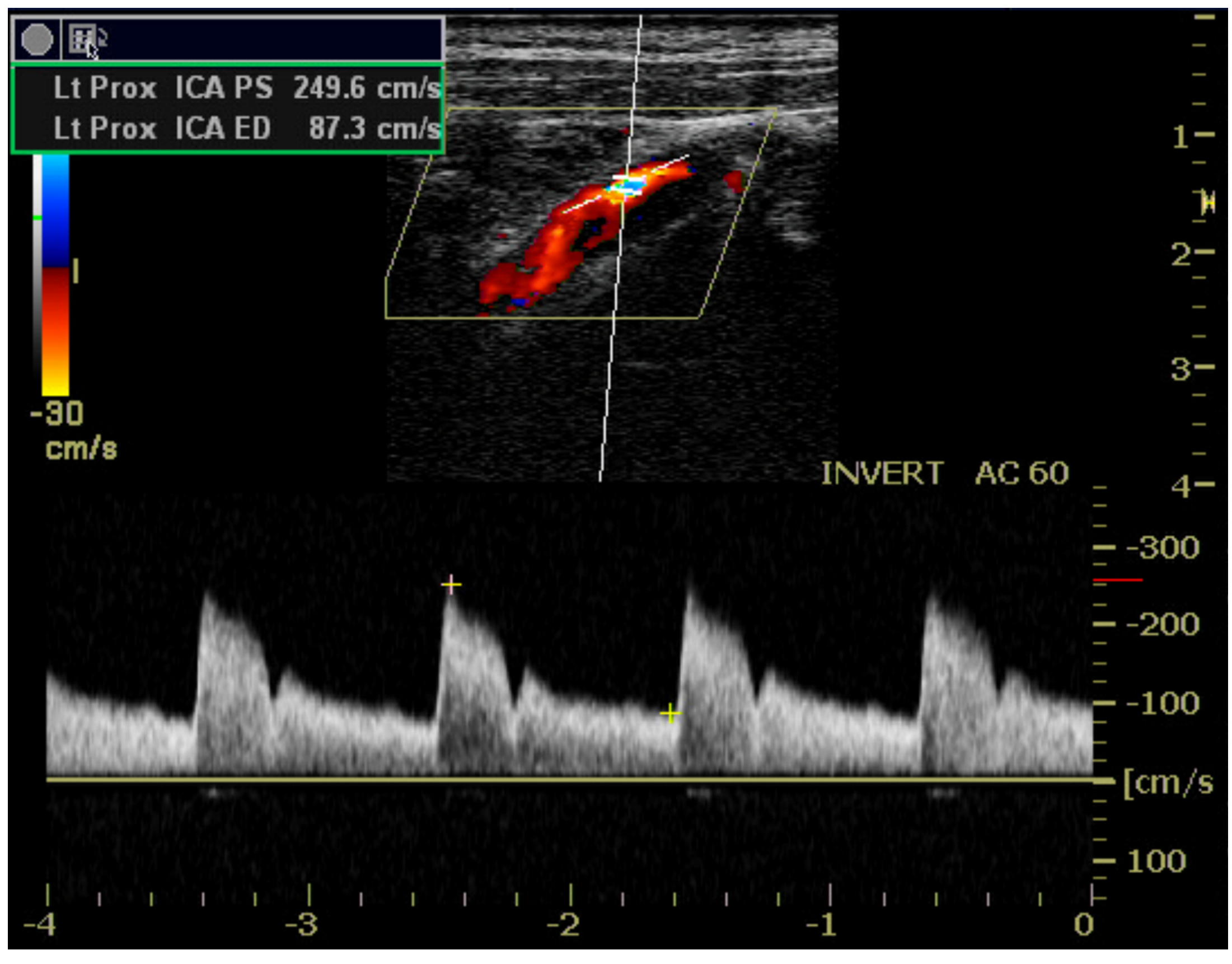

Supplement: Supplementary file 2 [file Image_1.TIF]
